# Targeting SARS-CoV-2 endoribonuclease: a structure-based virtual screening supported by in vitro analysis

**DOI:** 10.1038/s41598-022-17573-6

**Published:** 2022-08-03

**Authors:** Ibrahim M. Ibrahim, Abdo A. Elfiky, Mohamed M. Fathy, Sara H. Mahmoud, Mahmoud ElHefnawi

**Affiliations:** 1 Biophysics Department, Faculty of Science, Cairo University, Giza, 12613 Egypt; 2 Centre of Scientific Excellence for Influenza Viruses (CSEIV), National Research Centre, Cairo, 12622 Egypt; 3 Biomedical Informatics and Chemoinformatics Group (BICG), Informatics and Systems Department, National Research Centre, Cairo, 12622 Egypt

**Keywords:** Virtual screening, Computational biophysics

## Abstract

Researchers are focused on discovering compounds that can interfere with the COVID-19 life cycle. One of the important non-structural proteins is endoribonuclease since it is responsible for processing viral RNA to evade detection of the host defense system. This work investigates a hierarchical structure-based virtual screening approach targeting NSP15. Different filtering approaches to predict the interactions of the compounds have been included in this study. Using a deep learning technique, we screened 823,821 compounds from five different databases (ZINC15, NCI, Drug Bank, Maybridge, and NCI Diversity set III). Subsequently, two docking protocols (extra precision and induced fit) were used to assess the binding affinity of the compounds, followed by molecular dynamic simulation supported by the MM-GBSA free binding energy. Interestingly, one compound (ZINC000104379474) from the ZINC15 database has been found to have a good binding affinity of − 7.68 kcal/Mol. The VERO-E6 cell line was used to investigate its therapeutic effect in vitro. Half-maximal cytotoxic concentration and Inhibitory concentration 50 were determined to be 0.9 mg/ml and 0.01 mg/ml, respectively; therefore, the selectivity index is 90. In conclusion, ZINC000104379474 was shown to be a good hit for targeting the virus that needs further investigations in vivo to be a drug candidate.

## Introduction

The coronavirus belongs to the Coronaviridae family and the Riboviria realm, according to the International Committee on Virus Taxonomy (ICTV)^1^. It was discovered in humans for the first time in the 1960s, and there are currently seven strains of the virus that may cause illness in people. Out of them, four (OC43, HKU1, 229E, and NL63) produce moderate illness, while the other three (severe acute respiratory syndrome coronavirus (SARS-CoV), Middle-East respiratory syndrome coronavirus (MERS-CoV), and more recently, SARS-CoV-2) are more dangerous and can cause deadly diseases in humans^2^. SARS-CoV-2 has a 79% and 50% sequence similarity to SARS-CoV and MERS-CoV, respectively^3,4^. On the other hand, two bat CoV strains share a higher similarity with SARS-CoV-2 than that of the SARS-CoV. The bat coronavirus 'RaTG13' discovered in Rhinolophusaffinis is the closest relative to SARS-CoV-2 known to date, with 96.2% similarity. Following it is the bat coronavirus 'RmYN02,' which was recently discovered in a Rhinolophusmalayanus, with 93.3% similarity to the SARS-CoV-2 genome^5,6^. These two strains were discovered in Yunnan, a Chinese province. The new beta-coronavirus SARS-CoV-2 is a (+) sense RNA virus with a genome of nearly 30 kb. This genome encodes four structural genes: spike (S), envelope (E), membrane (M), and nucleocapsid (N) and nine accessory proteins (ORF3(a, b), ORF6, ORF7(a, b), ORF8, ORF9(b, c), and ORF10) which are responsible for the formation of the 16 Non-structural proteins (NSPs) numbered from 1 to 16^7,8^. Endoribonuclease (EndoU or NSP15) is a 346 residue protein with three domains namely, the N-terminal domain, middle domain, and the C-terminal domain. The N-terminal domain was found to be responsible for the formation of a hexamer, while the C-terminal domain contains the active site of the protein that facilitates replication and processing of sub-genomic RNAs^9–12^. It was found that NSP15 hydrolyzes the phosphodiester bond that is located at the uridine (U) sites of single and double-stranded RNA molecules. This cleavage produces 2′, 3′ cyclic phosphodiester and 5′ hydroxyl terminal^13^. Without the role of NSP15 in the replication of the SARS-CoV-2 virus, the host innate immune system would utilize the pattern recognition receptor MDA5 to recognize the PolyU sequence of the negative-sense viral RNA that is replicated from the poly Adenine sequence of the viral RNA^14^. This identification would lead to the RNA-activated antiviral response by inhibiting the formation of cytoplasmic stress granules with antiviral functions. Moreover, it has been reported that the deletion of NSP15 decreased viral replication significantly^15,16^. In addition, NSP15 was essential in interfering with interferon-beta (IFN-β) production and is associated with retinoblastoma tumor suppressor protein^17^. Based on its vital role in the replication of the virus, this protein is considered one of the potential targets of the SARS-CoV-2 virus.

Artificial neurons are used to process data in deep learning, a branch of machine learning. Text mining and picture pattern recognition are two sectors where deep learning has been used. The approach is also utilized to speed up the drug development processes in drug discovery, such as virtual screening, molecular docking, and QSAR models^18–21^. Deep learning (DL) algorithms have been used to predict liver injuries from drugs, detect coronavirus infection from radiography images, find new antibacterial compounds, and predict drug-target interactions^22–25^. DL in drug discovery requires the drugs to be vectorized before finding a mapping between these vectors and their properties. One of the advantages of deep learning in drug discovery is that it can be used to predict large-scale data quickly compared to the traditional ways^26^. Wang et al. have developed a deep learning model to screen drugs against NSP5 of SARS-CoV-2 using a directed message passing neural network. They first trained the model on experimental data of several beta CoVs, then fine-tuned it with newly discovered active and inactive drugs against SARS-CoV-2. The final model was used to screen approximately 5 million druglike compounds from the ZINC15 dataset. Moreover, they tested the top seven compounds, and one of them had a unique chemical backbone with an IC_50_ of 37.0 µM^26^.

In this study, we have targeted NSP15, the endoribonuclease, using five Different drug databases. A state-of-the-art Deep Learning library (Deep Purpose)^27^ was used to screen and filter the compounds before molecular docking, which was done in two steps using the Extra Precision (XP) mode and Induced Fit (IF) mode of the Glide tool under Schrödinger. This was followed by a triplicate Molecular Dynamic (MD) Simulation of 100 ns each. Then, the calculation of binding energy using the Molecular Mechanics-Generalized Born Surface Area (MM-GBSA) approach was done to find the binding strength of each compound to the receptor after MD Simulation and to find the contribution of each amino acid to the binding. In vitro assessments followed this to find its 50% inhibitory concentration (IC_50_), 50% cytotoxic concentration (CC_50_), and selectivity index (SI).

## Methods

### In silico methods

**Figure 1:**
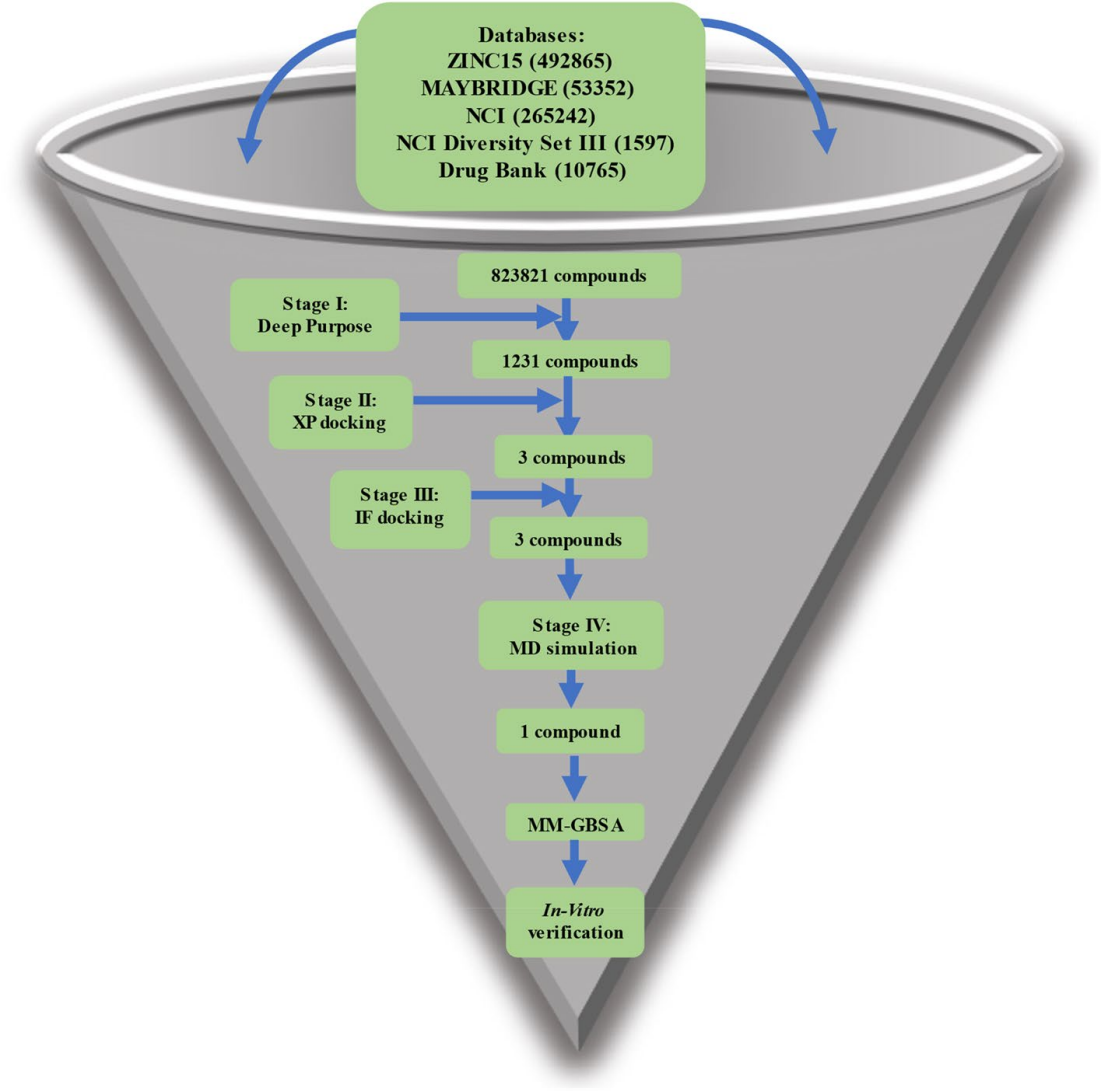
A workflow showing the steps performed in this study.

A workflow of analysis comprising six steps for structure-based drug design was adopted (Fig. 1). We utilized the Deep Purpose library to filter the compounds with predicted IC_50_ ≤ 100 nM. In the second step, we utilized the Glide tool in schrödinger to filter the compounds using XP and IF protocols which scored below (better) than the threshold of the redocked cocrystallized compound (citrate). In the third and fourth steps, we utilized the MD simulation to assert the binding of the remaining compounds. Then the MM-GBSA was calculated to find the binding energy and its components. The fifth and sixth steps included the clustering of the trajectory and the in vitro analyses to obtain IC_50_, CC_50_, and selectivity index.

#### Structural preparation

**Figure 2:**
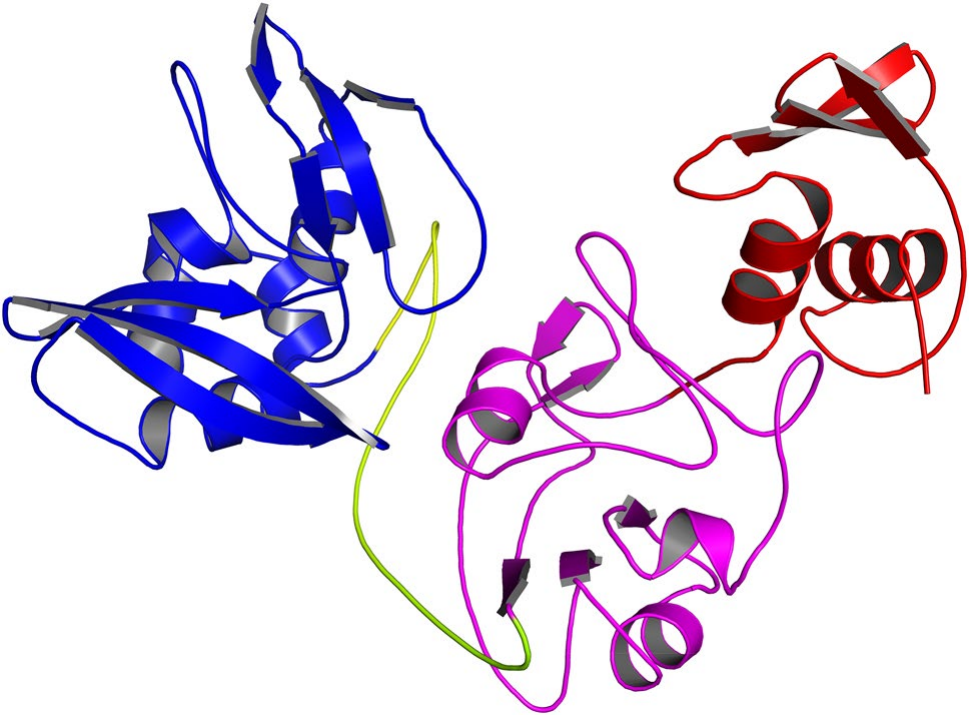
Shows the NSP15 domains. Red: The N-terminal domain. Magenta: The middle domain. Blue: The C-terminal domain which contains the active site of the protein.

Protein data bank (PDB) (https://www.rcsb.org/) was used to obtain the target protein (PDB ID: 6W01, Chain A) (endoribonuclease) as a FASTA sequence and 3D structure. First, unwanted atoms (water, co-crystallized ligand (citrate), polyethylene glycol, and ethylene glycol molecules) were removed from the structure before preparation using the protein preparation wizard in Maestro V 11.8 (Schrödinger). Next, missing side chains and loops were filled using PRIME, while missing Hydrogen atoms were added using the protein preparation wizard. In addition, PROPKA was used to generate protonation states at pH 7.0. Finally, a minimization step was initialized with 0.3 Å as a convergent metric for heavy atoms^28^. Figure 2 shows the 3D structure of the NSP15 after preparation and its three domains (N-terminal domain, Middle domain, and C-terminal domain). The total number of atoms was 5482 atoms.

Compounds in NCI Diversity set III (https://wiki.nci.nih.gov/display/ncidtpdata/compound+sets), NCI (https://cactus.nci.nih.gov/download/nci/), Drug Bank (https://go.drugbank.com/), Maybridge (http://www.maybridge.com/), and ZINC15 (https://zinc.docking.org/) (only drugs that are in stock and have 3D conformations) databases were downloaded for virtual screening (VS) using a python DL library called Deep Purpose^27^ and for docking using Maestro GLIDE^28^. For VS, Deep Purpose required the drugs to be in SMILES format; therefore, the drugs were converted into SMILES using open babel^29^ in the cases when the database did not provide a downloadable SMILES format. Deep Purpose has an encoder-decoder architecture with a total of 15 encoders for both the drugs and the proteins. In our case, we used Convolutional Neural Network (CNN), Morgan fingerprints, DayLight fingerprints, and Message passing neural network (MPNN) encoders to encode the drugs, while for the protein, we used a CNN and Amino Acid Composition (AAC) encoding. These encoders were combined (CNN_CNN, Daylight_AAC, Morgan_AAC, Morgan_CNN, MPNN_CNN; the first encoder is for the compound SMILES and the second is for the protein sequence) to generate five predictions. These predictions were further aggregated using mean_max aggregation $(\frac{{\text{value}}_{mean}+{\text{value}}_{\text{max}}}{2})$ to produce one value for each compound. The distribution of the SMILES lengths used in this study is shown in Supplementary Fig. S1 of Supplementary Material 1, which resembles the distribution of the SMILES used to train the models in the DeepPurpose library^27^. For the docking study, the 3D conformations of the drugs were downloaded. In the case of missing 3D structures, the 2D structure of the drugs was downloaded and then prepared using LigPrep in Maestro. Different ionization states were generated using Epik at a pH range of 7.0+/−2.0, and tautomers for each ionized or neutral compound were generated. For each drug, a maximum of 32 stereoisomers were generated.

#### Docking

The co-crystallized compound was redocked using GLIDE docking with the XP mode to get a docking score that will be used as a threshold for filtering drugs. Then, GLIDE docking with XP precision was used to dock the conformations of the prepared drugs to each protein, and the best results were selected using the score from the docking of the co-crystallized compound. The active site for endoribonuclease was H235, Q245, H250, K290, V292, T341, and Y343^30^. The default setting was used in XP docking. After that, an IF docking for the best compounds from XP docking was performed using the default settings and the same active site for each protein.

#### MD simulation

MD simulation on the best three complexes (ZINC000104379474, ZINC000004715217, ZINC000408720658) from the IF docking was done as a triplicate using NAMD for 100 ns each to make sure that the results we obtain are not a coincidence or caused by the artificial forcefield used^31^. The systems were prepared using the input generator module in the CHARMM-GUI web server^32–35^. For each complex, the system was solvated in the TIP3P water model. The temperature was set to 310 K, and the system was neutralized by adding Na^+^ and Cl^-^ ions with a concentration of 0.154 M. Furthermore, the periodic boundary conditions (PBC) were applied during the simulation. Each system was minimized for 10,000 steps using the Conjugate Gradient algorithm, and the time step was set to 2 femtoseconds. Following minimization, an equilibration step for a nanosecond in a constant number of atoms, constant pressure, and constant temperature (NPT) ensemble was performed. Temperature and pressure were maintained at 310 K and 1 atm using Langevin dynamics and Nose–Hoover Langevin piston. Finally, a production run of 100 ns was performed in a constant number of atoms, constant volume, and constant temperature (NVT) ensemble.

#### Molecular mechanics-generalized born surface area (MM-GBSA)

Gmx_MMPBSA^36^ was used to calculate the binding affinity using MM-GBSA with interaction entropy. The salt concentration was adjusted to 0.154 M, and the method of generalized born (igb) was set to 5. Additionally, decomposition of the free energy was obtained to determine the contribution of amino acids within 1 nm of the ligand.

#### Clustering and finding interactions

TTClust python library was used to cluster the trajectories and obtain a representative frame for each cluster. Alignment was performed first on the backbone before clustering, and the number of clusters was determined automatically using the elbow method^37^. Protein–Ligand Interaction Profiler (PLIP) was used with each representative frame to find the number and types of interactions^38^.

### In vitro methods

#### Cytotoxicity (CC_50_) determination

The compound that showed a good result in MD simulation was continued for in vitro studies to assess its therapeutic effect. The compound was obtained from NCI and dissolved in dimethyl sulfoxide (DMSO, Sigma-Aldrich) at a 1 mg/ml concentration and stored at 4 °C. To assess the CC_50_, the stock solution of the compound was diluted further to the working solutions with DMEM (1–0.001 mg/ml). Cytotoxic activity was tested in VERO-E6 cells using a crystal violet assay as previously described by Feoktistova et al.^39^ with minor modifications. Briefly, the cells were seeded in 96-well plates (100 μl/well at a density of 3 × 10^5^ cells/ml) and incubated for 24 h at 37 °C in 5% CO_2_. After 24 h, the cells were treated with various concentrations of the compound in quadruplicate. At 72 h post-treatment, the supernatant was discarded, and cell monolayers were fixed with 10% formaldehyde for 1 h at room temperature (RT). The fixed monolayers were then dried thoroughly and stained with 50 μl of 0.1% crystal violet for 20 min on a bench rocker at room temperature. The monolayers were then washed and dried overnight. The crystal violet dye in each well was dissolved in 200 μl methanol for 20 min on a bench rocker at room temperature. The absorbance of the crystal violet solutions was measured at λ_max_ 570 nm as a reference wavelength using a multi-well plate reader. The CC_50_ value was calculated using nonlinear regression analysis using GraphPad Prism software (version 5.01) by plotting log concentrations of the compound versus normalized response (variable slope).

$$\text{cytotoxicity \%}=\frac{\text{the absorbance of cells without treatment}-\text{the absorbance of cells with treatment}}{\text{the absorbance of cells without treatment}}\times 100$$

The concentration that displayed CC_50_ was calculated using a plot of percent cytotoxicity vs sample concentration as previously described by Mosmann^40^.

#### *Inhibitory concentration 50 (IC*_*50*_*) determination*

The IC_50_ value for the compound was determined as previously described^41^, with slight modifications. 2.4 × 10^4^ Vero-E6 cells were placed in each well of 96-well tissue culture plates and cultured overnight at 37 °C in a humidified 5% CO_2_ incubator. After that, the cell monolayers were rinsed once in 1 × PBS. An aliquot of the SARS-CoV-2 "NRC-03-nhCoV" virus^42^ containing 100 TCID_50_ was incubated with serially diluted concentrations of the tested compound and kept at 37 °C for 1 h. Another set of Vero-E6 cells was treated with virus/compound mix and co-incubated at 37 °C in a total volume of 200 µl per well. Untreated cells infected with a virus represent virus control; however, cells that have not been treated and have not been infected represent cell control. The cells were fixed with 100 μl of 10% paraformaldehyde for 20 min and stained with 0.5% crystal violet in distilled water for 15 min at room temperature after being incubated for 72 h at 37 °C in a 5% CO_2_ incubator. After that, 100 μl absolute methanol per well was used to dissolve the crystal violet dye, and the optical density of the color was measured at 570 nm using an Anthos Zenyth 200rt plate reader (Anthos Labtec Instruments, Heerhugowaard, Netherlands). The IC_50_ of the compound is that required to reduce the virus-induced cytopathic effect (CPE) by 50%, relative to the virus control. The IC_50_ value was calculated using nonlinear regression analysis of GraphPad Prism software (version 5.01) by plotting log concentrations of ZINC000104379474 versus normalized response (variable slope).

## Results

### Screening results

All compounds that were downloaded from different databases, a total of 823,821compounds, were converted into SMILES format for screening using DeepPurpose with the NSP15. The results from the library are continuous values indicating the binding score. The best compounds were selected using a threshold of 100 nM, which resulted in 1231 compounds for NSP15 (Supplementary Material 2). These compounds were prepared for docking, as mentioned in the methods section.

### Docking results

**Figure 3:**
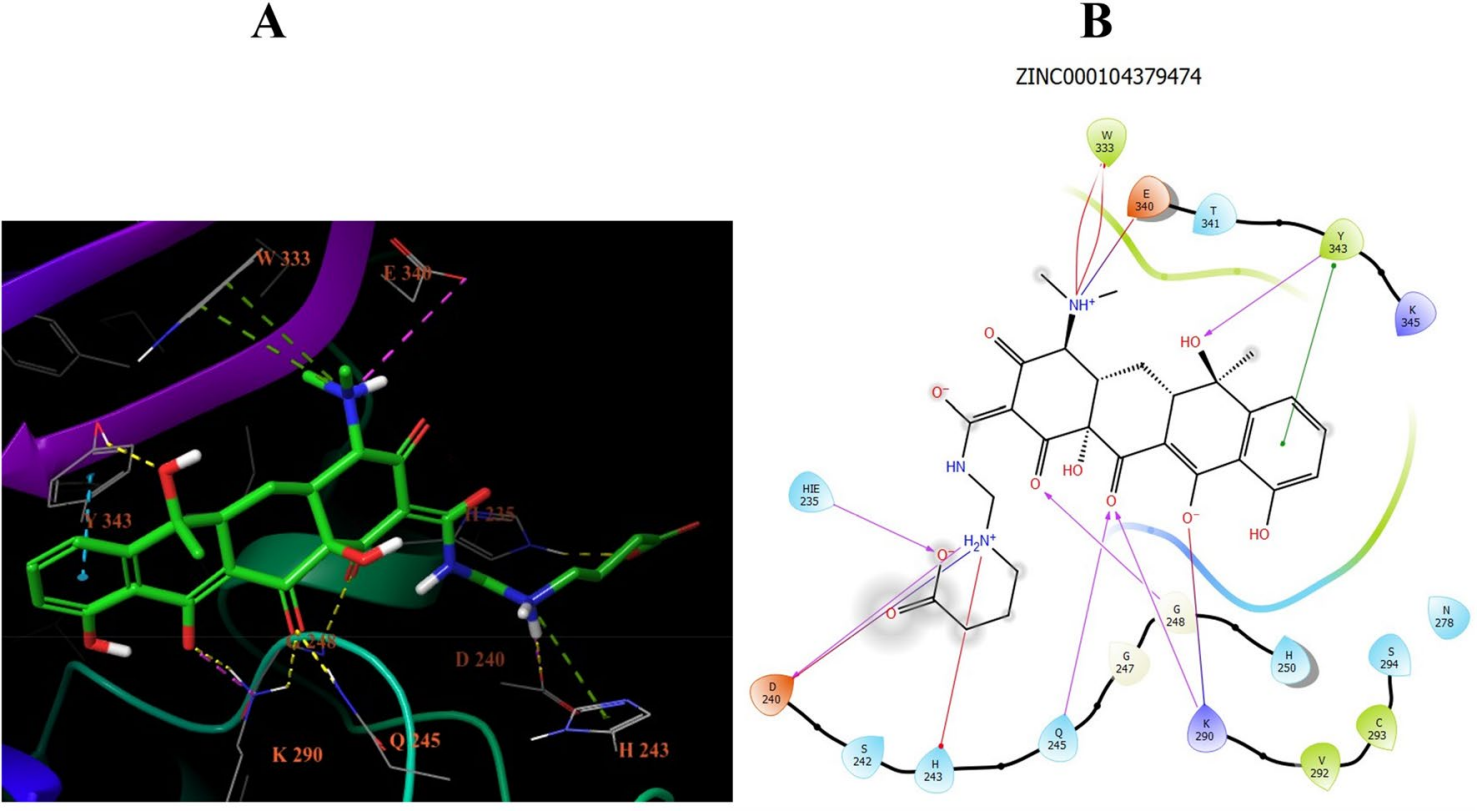
The 2D and 3D interactions between the IF docking of ZINC000104379474 and NSP15. There are 12 interactions in total. Half of them (six interactions) are H-bonds between H235, D240, Q245, G248, K290, and Y343, followed by three salt bridges (D240, K290, and E340), then one Pi-cation (H243) interaction and two Pi–Pi stacking (Y343).

Before the XP docking of prepared compounds, a redocking step was performed with the co-crystallized compound to get a threshold docking score to filter the docking results of the prepared compounds. The threshold was − 7.225 kcal/mol for NSP15. After filtering the XP docking results, three compounds were found to have a docking score better (more negative) than the threshold values. These compounds were further filtered using IF docking mode based on the same docking threshold from the redocking step. This produced the same three compounds for NSP15, and they were selected for MD simulation. Their IDs are ZINC000104379474 (73 atoms), ZINC000004715217 (71 atoms), and ZINC000408720658 (53 atoms). The 2D interactions from the IF docking of ZINC000104379474 with the NSP15 is shown in Fig. 3. The interaction consists mostly of H-bonds (six interactions) with H235, D240, Q245, G248, K290, and Y343. In addition, there are three salt bridges (D240, K290, and E340). Moreover, two Pi-Pi stacking interactions emerged with Y343 and a Pi-Cation interaction with H243.

### MD simulation results

**Figure 4:**
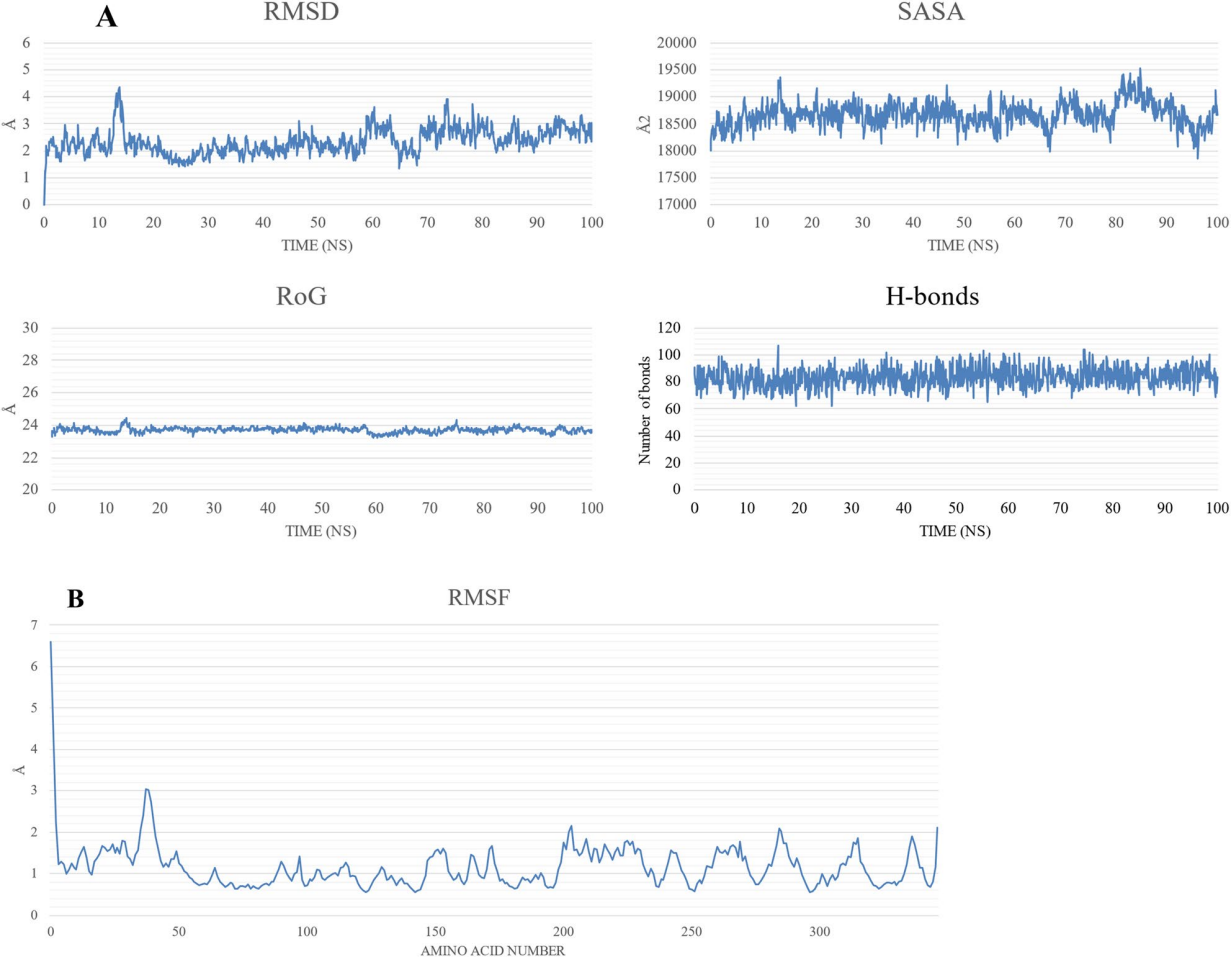
The molecular dynamics simulation analysis of the NSP15-ZINC000104379474 complex. (**A**) The radius of gyration (RoG) in Å, root-mean-square displacement (RMSD) in Å, surface accessible surface area (SASA) in Å^2^, and the total number of H-bonds versus the simulation time in ns. (**B**) The per-residue root-men-square fluctuation (RMSF) in Å.

NAMD was used to perform the MD simulation of the best three complexes. For NSP15-ZINC000004715217 and NSP15-ZINC000408720658, the ligands were found to dissociate from the binding site during the production run for two replicates out of three (at 10 and 88 ns, respectively, for the first replicate and 43 and 58 ns, respectively, for the second replicate); therefore, these complexes were excluded from further analysis. On the other hand, ZINC000104379474 remained bound to NSP15 for the whole duration of the simulation in two out of the three replicates. Only the first replicate’s results are reported in the main text, while the second replicate’s results can be found in the Supplementary Material 1 file (Supplementary Figs. S2–S4, and Supplementary Tables S1 and S2). The NSP15-ZINC000104379474 complex shows average values of 23.7 Å, 2.34 Å, 18,669 Å^2^, and 83 for the radius of gyration (RoG), root-mean-square displacement (RMSD), surface accessible surface area (SASA), and the total number of H-bonds, respectively, during the 100 ns simulation period as depicted in Fig. 4A. The stability of SASA, RoG, and H-bonds indicates the stability of the NSP15-ZINC000104379474 system. The RMSD values indicate system equilibration over the trajectory. The first 58 ns show a stable fluctuation except for the duration from 12.5 to 15 ns which shows a spike reaching above 4 Å before returning to stability. The RMSF plot (Fig. 4B) shows small fluctuations except for the N-terminal region (RMSF < 6.5 Å).

**Figure 5:**
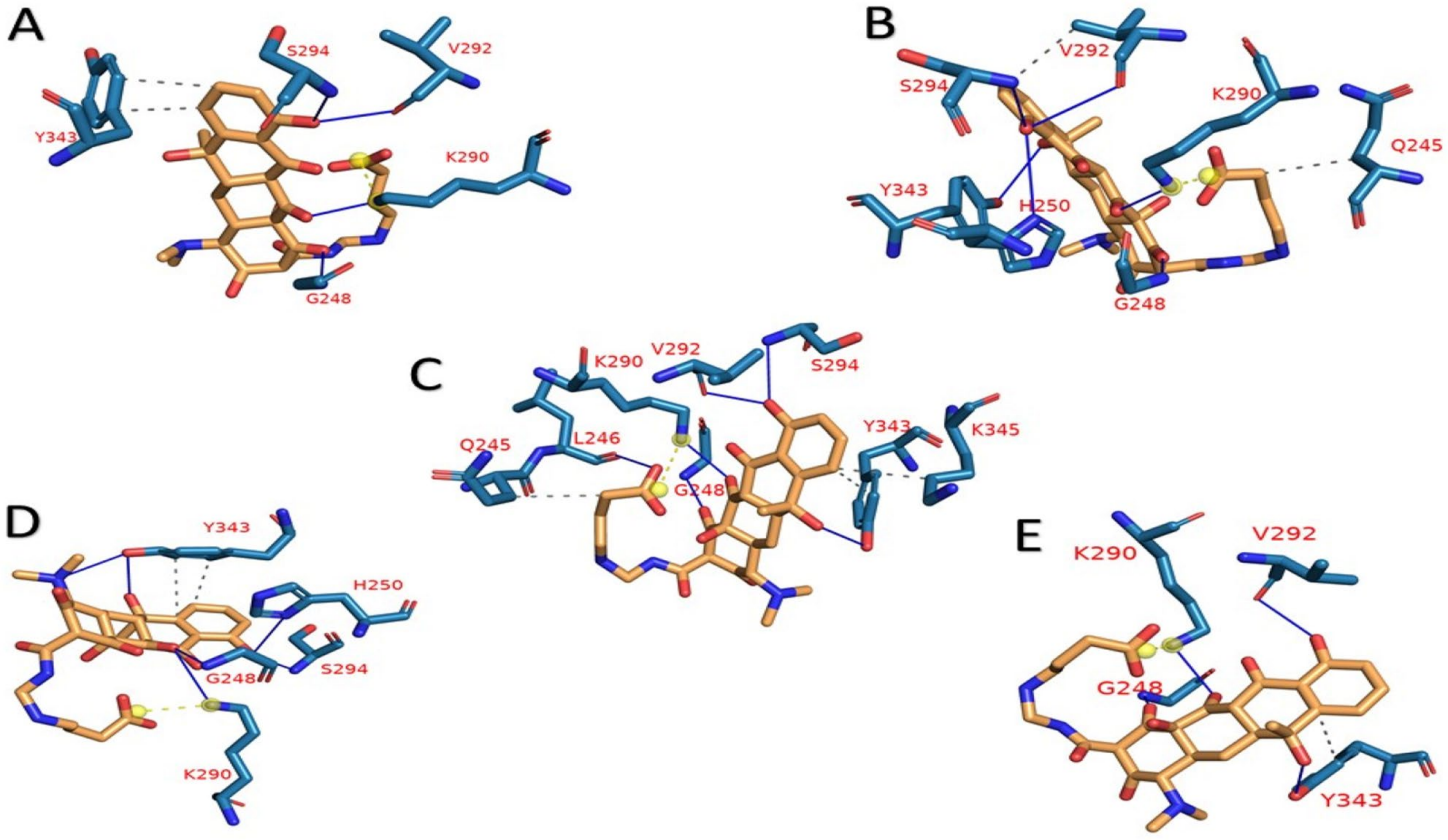
the representative frames (**A**–**E**) from clustering using TTClust, and the types and number of interactions calculated and depicted using PLIP webserver for NSP15-ZINC000104379474 complex. Dashed-Grey lines: Hydrophobic interactions, blue lines: H-bond, dashed-yellow lines: salt-bridges.

**Figure 6:**
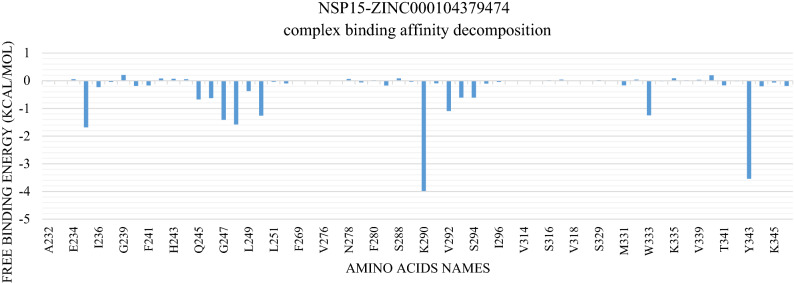
Molecular Mechanics Generalized Born Surface Area (MM-GBSA) decomposition of NSP15-ZINC000104379474 complex. It shows the contribution of amino acids within 1 nm of the ZINC000104379474 compound.

Figure 5 and Table 1 show the interactions in each cluster representative with ZINC000104379474 that were obtained from the PLIP webserver. The interaction between ZINC000104379474 and NSP15 is primarily by H-bonding. G248 and K290 are the most common residues that contribute to drug-protein interaction in all clusters, followed by V292, S294, and Y343. The most common amino acid responsible for hydrophobic contacts is Y343. The binding free energy calculation using MM-GBSA shows that the NSP15-ZINC000104379474 complex has an average binding energy of − 7.68 kcal/mol. The decomposition of binding energy for the NSP15-ZINC000104379474 complex (Fig. 6) shows that amino acids H235, G247, G248, H250, K290, V292, W333, Y343 have binding affinity of − 1.68, − 1.41, − 1.58, − 1.25, − 3.98, − 1.09, − 1.25, − 3.54 kcal/mol, respectively. Table 2 shows the different energy terms calculated by MM-GBSA and the binding energy.

**Table 1:** PLIP web server analysis for NSP15-ZINC000104379474 complex.

Cluster	Number of hydrogen bonds	Amino acids in NSP15	Number of hydrophobic interactions	Amino acids in NSP15	Number of salt bridge interactions	Amino acids in NSP15
A	4	**G248**–**K290**–V292–S294	2	Y343 (2)	1	**K290**
B	6	**G248**–H250–**K290**–V292–S294–Y343	2	Q245–V292	1	**K290**
C	6	L246–**G248**–**K290**–V292–S294–Y343	3	Q245–Y343–K345	1	**K290**
D	6	**G248**–H250–**K290**–S294–Y343 (2)	2	Y343 (2)	1	**K290**
E	4	**G248**–**K290**–V292–Y343	1	Y343	1	**K290**

Bold amino acids indicate that they are most common in all cluster representatives.

**Table 2:** Binding free energies and individual energy terms and their standard deviation of NSP15-ZINC000104379474 complex calculated by MM-GBSA. Units are in kcal/mole.

System	∆E_VDW_	∆E_ELE_	∆G_GB_	∆G_SA_	∆G_GAS_	∆G_SOLV_	∆G_TOTAL_	− T∆S	∆G_BINDING_
NSP15-ZINC000104379474 complex	− 31.08 ± 3.68	− 251.31 ± 15.34	260.34 ± 14.42	− 4.90 ± 0.29	− 282.39 ± 14.63	255.44 ± 14.35	− 26.95 ± 5.18	19.28 ± 2.1	− 7.68 ± 5.59

### ADMET prediction

The Absorption, Distribution, Metabolism, Excretion, and Toxicity (ADMET) properties for ZINC000104379474 were predicted using the pkCSM web server^43^. Table 3 shows the predicted values for each property. ZINC000104379474 shows a water solubility value of − 2.899 log (mol/l) which is in the same region as other druggable compounds^44^. The fraction unbound indicates the predicted fraction of the compound that will be unbound to serum proteins. High unbound fraction and low Blood–Brain Barrier (BBB) permeability (log BBB) indicate a good distribution. Inhibitors of Cytochrome P450 can activate the drug metabolism and, therefore, can remove the compound from the market. ZINC000104379474 compound was used to predict whether it is an inhibitor for different isoforms (CYP1A2, CYP2C19, CYP2C9, CYP2D6, and CYP3A4). The server predicted that the compound is not an inhibitor for any of the isoforms. For Excretion, the model predicts that the compound is not a substrate for renal organic cation transporter 2. The interaction with this transporter helps in the clearance of the compound and may produce adverse interactions; therefore, a negative prediction is considered good. Finally, four indicators are used to predict the toxicity of ZINC000104379474. Ames toxicity is a test that indicates whether the compound is a carcinogen. Inhibition of hERG I/II is the principal cause of fatal ventricular arrhythmia and has resulted in the withdrawal of many substances. As its name implies, Hepatotoxicity indicates whether the compound may disrupt the liver's normal function. Fortunately, the server predicts that the compound does not cause any toxicity.

**Table 3:** pkCSM webserver analysis of ADMET properties for ZINC000104379474 compound.

Property	Model name	Predicted value
Absorption	Water solubility	− 2.899
Distribution	Fraction unbound (human)	0.353
	Blood–brain barrier permeability	− 1.676
Metabolism	CYP1A2 inhibitior	No
	CYP2C19 inhibitor	No
	CYP2C9 inhibitor	No
	CYP2D6 inhibitor	No
	CYP3A4 inhibitor	No
Excretion	Renal OCT2 substrate	No
Toxicity	AMES toxicity	No
	hERG I inhibitor	No
	hERG II inhibitor	No
	Hepatotoxicity	No

### In vitro results

**Figure 7:**
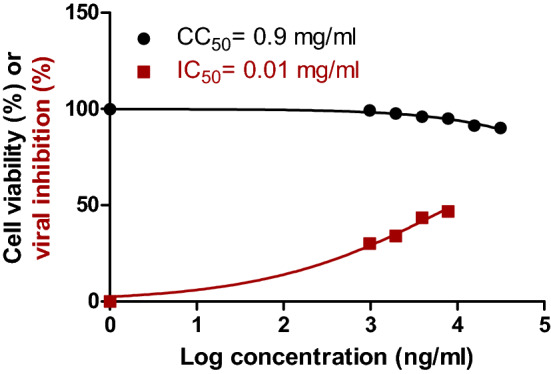
Dose–response and inhibition curves for ZINC000104379474 compound on VERO-E6 cells. Half maximal cytotoxic concentration (CC_50_) in Vero E6 cells (black) and inhibitory concentration 50% (IC_50_) against NRC-03-nhCoV were calculated using nonlinear regression analysis of GraphPad Prism.

The tested sample of the ZINC000104379474 compound against the SARS-CoV-2 virus in VERO E6 cells showed IC50 and CC50 values of 0.01 and 0.9 mg/ml (Fig. 7), respectively. Therefore, its selectivity index is 90. Since the CC50 value is 90-fold greater than the IC50, this compound shows a potential to be used against the SARS-CoV-2 virus. (CC50 > IC50; SI > 1 (90)).

## Discussion

Since the emergence of SARS-CoV-2, many research groups worldwide have started searching for drugs that can affect the virus life cycle utilizing different in silico techniques ranging from classical virtual screening and molecular docking to the usage of state-of-the-art deep learning models. Some of the recent work done by other research groups to find new inhibitors for SARS-CoV-2 proteins is mentioned here. El-Demerdash, A. et al. have screened 15 guanidine alkaloids against two NSPs (NSP10 and NSP5) and three structural proteins (S–N–M), calculated their binding affinities, determined their structure–activity relationship (SAR), and predicted their Absorption, Distribution, Metabolism, Excretion, and Toxicity properties. Two of the screened compounds (crambescidin 786 and crambescidin 826) showed promising results against their target proteins^45^. El Hassab et al. have screened 48 million druglike compounds obtained from the ZINC database against NSP16 using a pharmacophore-based screening which resulted in only 24 compounds. These compounds were subsequently filtered into four based on the target protein’s docking scores before running an MD simulation. Their results showed that only one compound had a good binding affinity compared to the pan-methyl-transferase inhibitor Sinefungin and might act as an inhibitor for NSP16^46^. Alazmi et al. have screened approximately 100 thousand natural compounds from the ZINC database against NSP4, NSP15, RdRp, and ACE2, followed by docking and MD simulation. Their work showed that Baicalin could bind to the three viral proteins, while Limonin can bind ACE2, rendering it inefficient in binding SARS-CoV-2 RBD^47^. Elgohary et al. targeted the main protease of SARS-CoV 2 virus with 15 batzelladine marine alkaloids. They started their approach by performing docking followed by MD simulation and SAR^48^. Kumar et al. have performed high throughput screening of an in-house database against NSP15 followed by MD simulation for the high-ranked compounds. A derivative of piperazine was found to bind to the active site of NSP15 and demonstrated inhibition of viral replication by in vitro assays^2^. Mahmud et al. have performed a repurposing study of 23 compounds against NSP15 and found three compounds with a predicted binding affinity better than − 9.0 kcal/mol^17^. Savale et al. have screened 8722 small molecules from the Asinex antiviral database against NSP15 protein. They prepared the compounds and the protein using the LigPrep module in the Schrödinger suite and protein preparation wizard before docking using a multi-step docking workflow. The top-ranked molecules were selected based on the Prime-MMGBSA approach and Glide-XP score. The top five molecules were then checked for druglike properties and toxicity using SwissADME and PkCSM webservers. Finally, they performed MD simulation for the complexes and calculated binding free energy using the MM-PBSA approach and found that five compounds showed strong binding affinity^49^.

Nidoviral RNA uridylate-specific endoribonuclease (NendoU or NSP15) is one of the crucial 16 NSPs of SARS-CoV-2. Its C-terminal has a catalytic site specific to uridine. It was recently discovered that NSP15 has a role in the innate immune response, and therefore it is considered an important target for combatting the infection^50,51^. NSP15 consists of the N-terminal, middle, and catalytic C-terminal domains. It specifically cuts the double-stranded RNA through an Mn^+2^-dependent activity. The C-terminal domain consists of two antiparallel β-sheets that hold six key amino acids (H235, H250, K290, S294, T341, and Y343)^51^.

In this work, we performed a multi-database virtual screening with 823,821 compounds using a Deep Learning approach followed by docking using the XP and IF docking implemented in Schrödinger Suite^28^. Only 1231 compounds were selected from the results of the deep learning library based on a threshold of 100 nM. After their preparation using LigPrep in Maestro, 1314 different conformations were obtained. These conformations were docked using the XP method, and the top compounds (3 compounds) were selected for the IF docking protocol. The compounds with a better docking score (3 compounds) than that of the co-crystalized drug were selected for the subsequent analyses. The three molecules were selected for three replicates of 100 ns MD simulation each. After removing the PBC, the trajectory was analyzed for its stability using the TK console in VMD^52^, then the binding affinity with entropy was calculated using the MM-GBSA approach in gmx_MMPBSA^36^. Figure 4 shows the plots of the analyses done on the trajectories.

Furthermore, the trajectories were clustered to obtain representative frames using TTClust^37^. These frames were used with PLIP to detect the types of interactions between NSP15 and ZINC000104379474^38^. This compound was found to interact with most of the key amino acids responsible for the catalytic activity of NSP15 with an average binding affinity of − 7.68 kcal/Mol. H250 forms one H-bond in just two clusters with − 1.25 kcal/mol contribution to the total binding energy. Besides, K290 is a common amino acid among the five clusters used to analyze the interaction. It interacts with ZINC000104379474 via one H-bond and one salt bridge in each cluster. It shows the most substantial binding among the other amino acids with a − 3.98 kcal/mol contribution. On the other hand, S294 interacts with ZINC000104379474 through both H-bond and hydrophobic interaction with only − 0.6 kcal/mol in the first four clusters. Additionally, Y343 forms two types of interactions with ZINC000104379474. In cluster 1, it forms one hydrophobic interaction, while in the second cluster, it forms one H-bond. In addition, it forms both types of interaction in the last three clusters, with only four interactions (two H-bonds and two hydrophobic interactions) in cluster 4. It has a contribution of -3.54 kcal/mol to the total binding affinity. Although H235 is not found to have any interaction with the compound in these five clusters, it has a contribution of − 1.68 kcal/mol. On the other hand, T341 has a minimal contribution of only − 0.16 kcal/mol. Moreover, G248 forms one hydrogen bond in all cluster representatives with a contribution of − 1.58 kcal/Mol. In addition to this, V292 forms one hydrogen bond in four out of five cluster representatives with a contribution of − 1.09 kcal/Mol. Although G247 and W333 were not found to form any interaction in the cluster representatives, they have a contribution of − 1.41 kcal/Mol, and − 1.25 kcal/Mol, respectively.

Based on the in silico, the CC_50,_ and the IC_50_ results, ZINC000104379474 is expected to have potent inhibitory activity against SARS-CoV-2 NSP15. Therefore, it is recommended to be used as an antiviral agent after in vivo validation.

SARS-CoV-2 is still affecting our daily lives despite many vaccine shots. Therefore, an urgent need for possible therapeutics is mandatory to inhibit the viral spread. In this study, we screened five different databases for possible anti-SARS-CoV-2 endoribonuclease. The compounds are then filtered using different computational methods giving us three compounds that bind the NSP15. The MDS further select the compound ZINC000104379474 as the best potential NSP15 inhibitor. The in vitro study further validates this result as the ZINC000104379474 has IC_50_ and CC_50_ values of 0.01 and 0.9 mg/ml. Further in vivo validation is suggested as future work.

## Supplementary Information

Supplementary Information 1.

Supplementary Information 2.

## Data Availability

Data is available upon request from the corresponding author.
