# Supplementary material for: Targeting SARS-CoV-2 endoribonuclease: a structure-based virtual screening supported by in vitro analysis
Source: Sci Rep. 2022 Aug 3;12:13337. doi: 10.1038/s41598-022-17573-6 (PMC9349323; doi:10.1038/s41598-022-17573-6)
Supplement: Supplementary file 1 — Supplementary Information 1. [file 41598_2022_17573_MOESM1_ESM.pdf]

Supplementary material 1

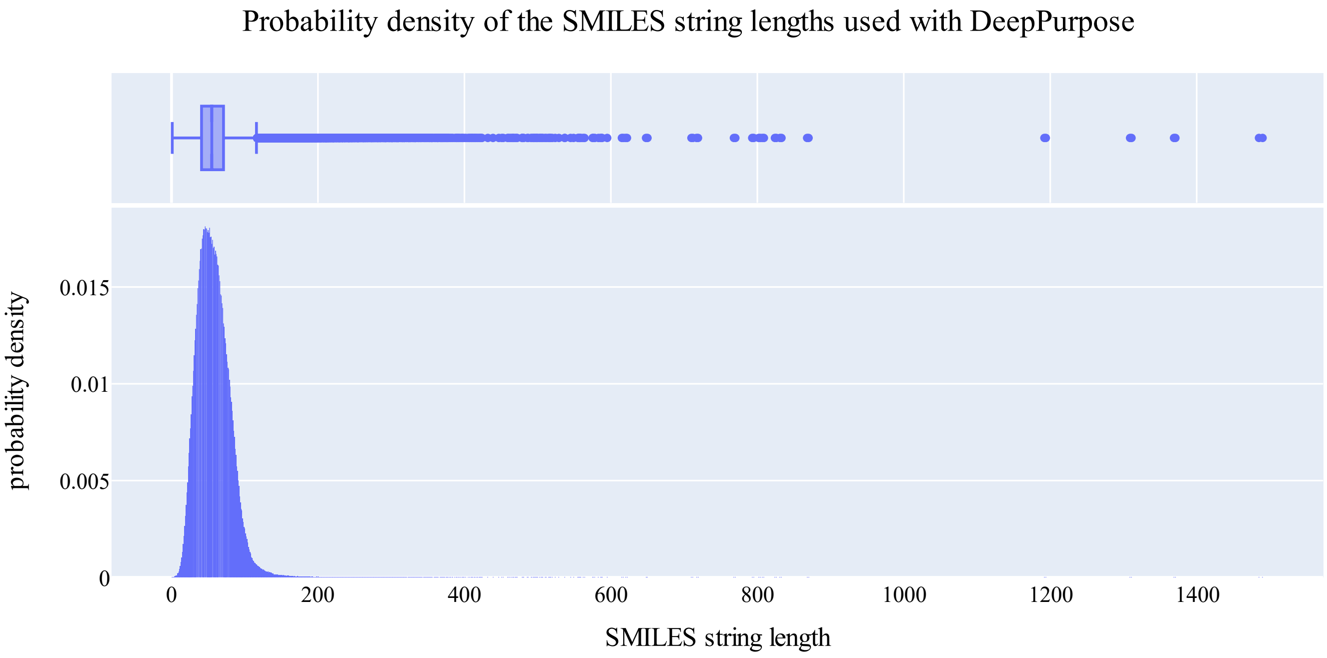

Supplementary Figure S1 *The distribution of the SMILES lengths of compounds used in this study with the DeepPurpose library*

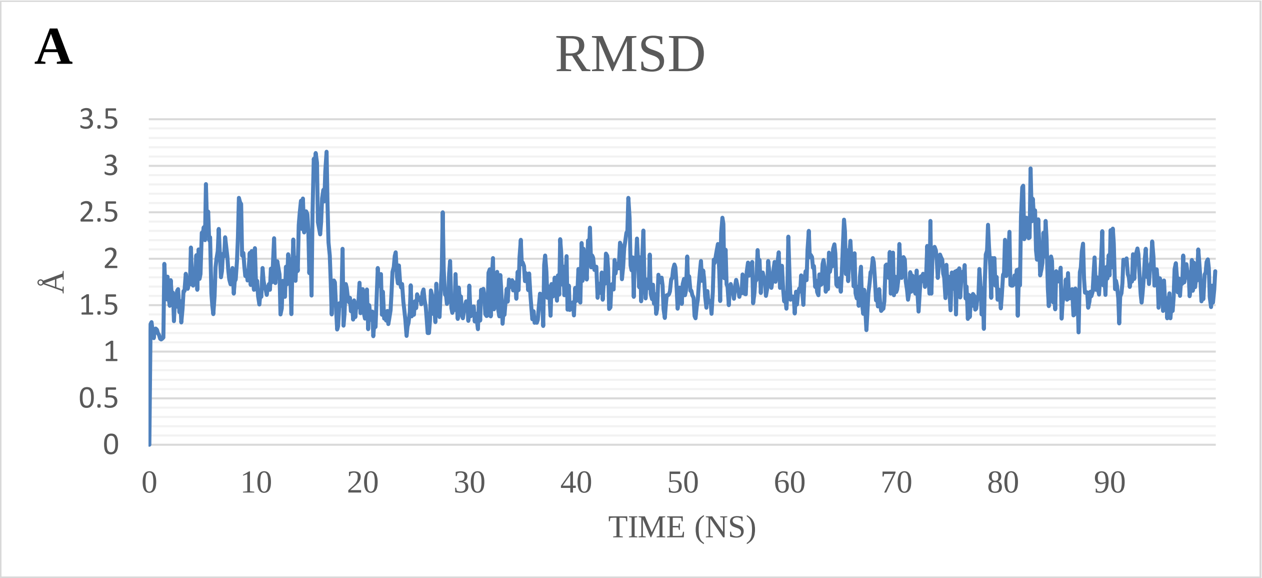

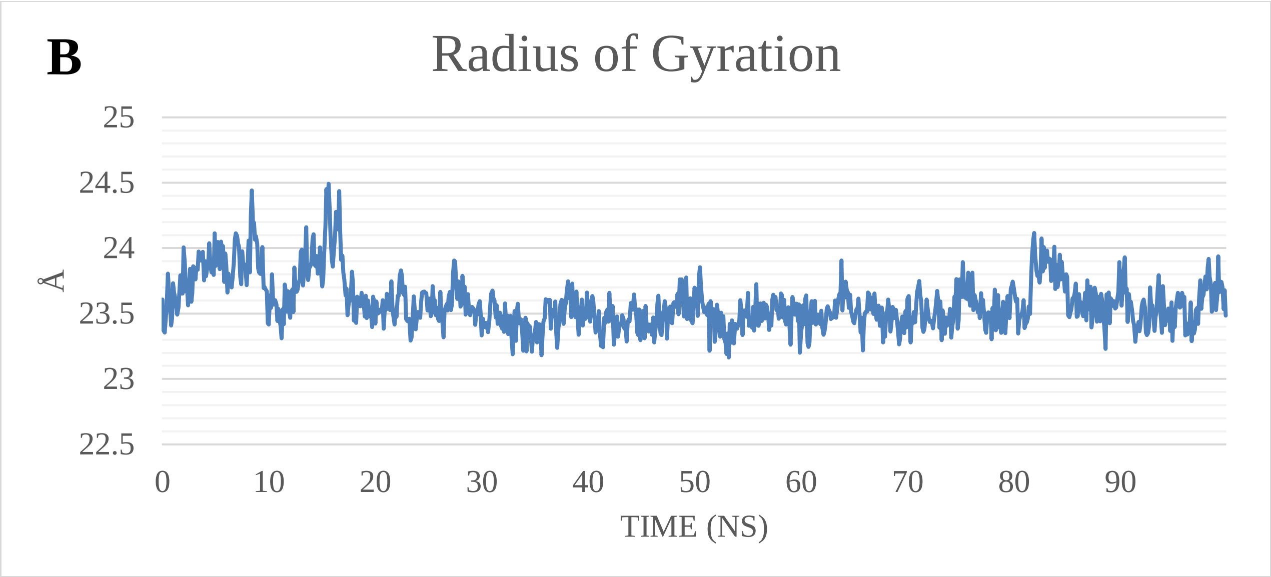
B
Radius of Gyration
25
24.5
24
23.5
23
22.5
Å
0
10
20
30
40
50
60
70
80
90
TIME (NS)

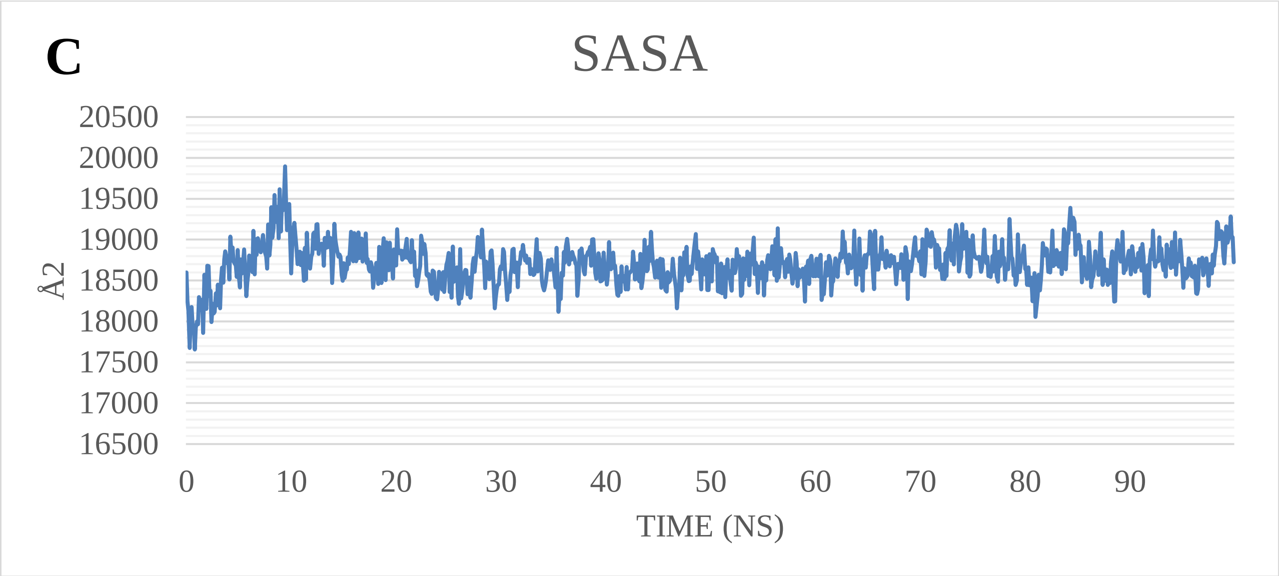
C
SASA
20500
20000
19500
19000
18500
18000
17500
17000
16500
Å2
0
10
20
30
40
50
60
70
80
90
TIME (NS)

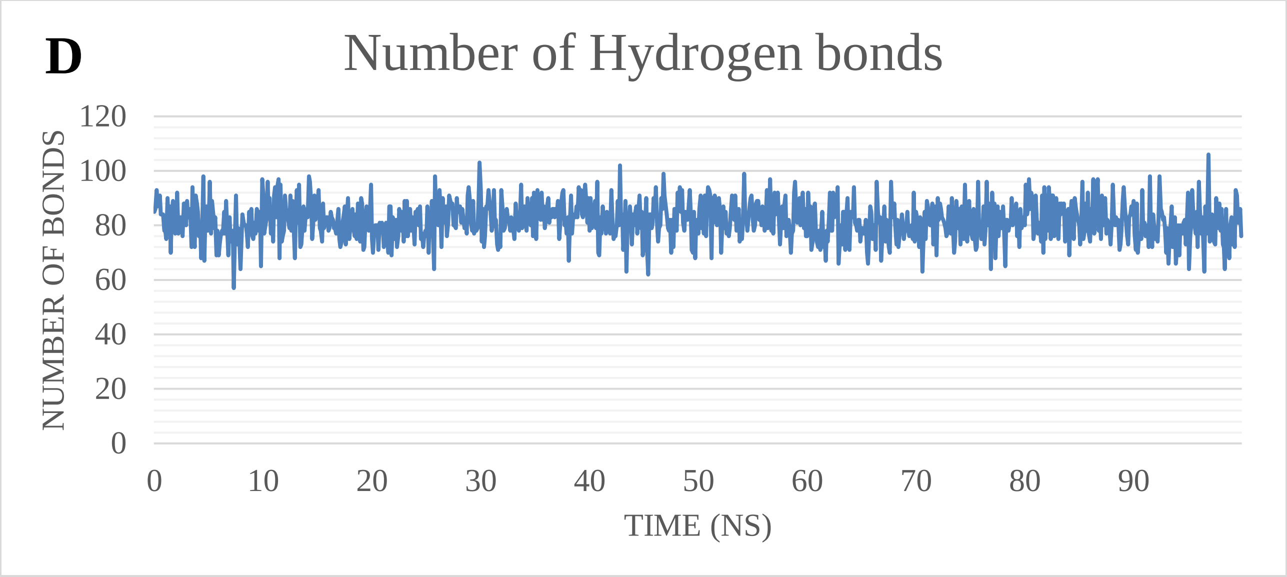

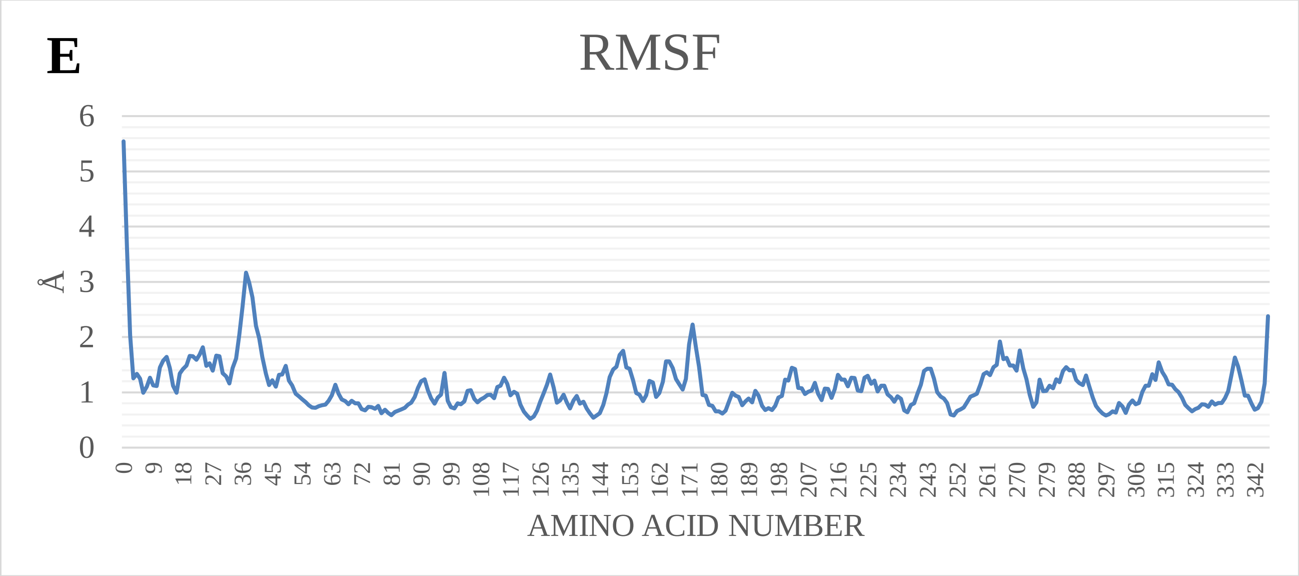

Supplementary Figure S2 *shows the analyses done on the second replicate of the ZINC000104379474-NSP15 complex. A) RMSD shows an average value of 1.76 Å. The values show equilibration after approximately 17 ns. B) radius of gyration shows an average value of 23.58 Å with a similar trend to the RMSD. C) the SASA values show an increase in the first 10 ns before stabilizing at an average of 18710 Å^2. D) the number of hydrogen bonds shows an average value of 82 bonds. E) the RMSF values show small fluctuations for nearly most of the amino acids except for the N-terminal (reaching 5.5 Å).*

Supplementary table S1 *PLIP web server analysis for NSP15-ZINC000104379474 complex second replicate.*

CLUSTER NUMBER	NUMBER OF HYDROGEN BONDS	AMINO ACIDS IN RECEPTOR	NUMBER OF HYDROPHOBIC INTERACTIONS	AMINO ACIDS IN RECEPTOR	NUMBER OF SALT BRIDGES	AMINO ACIDS IN RECEPTOR
**C1**	4	H250 - K290 - S294 - T341	3	Q245 - V292 - Y343	1	K290
**C2**	5	G248 - H250 - K290 - S294 - T341	2	V292 - Y343	2	H235 - K290

C1

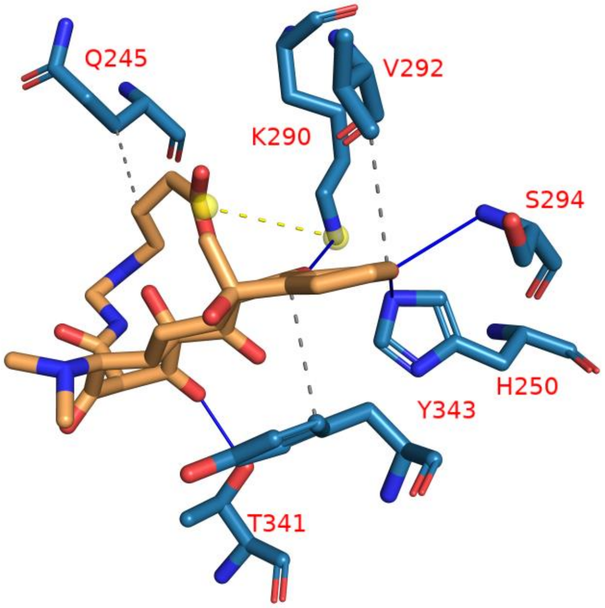

C2

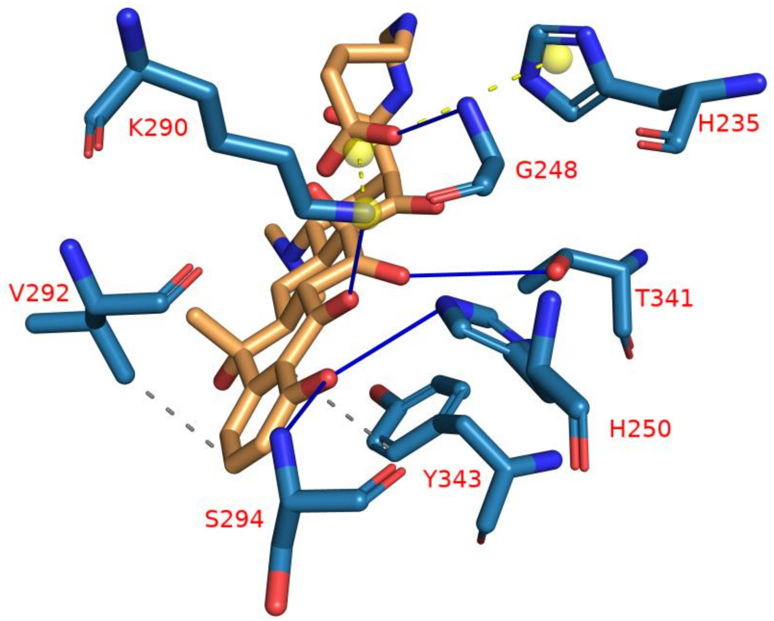

***Supplementary Figure S3** shows the interactions detected using PLIP between ZINC000104379474 and NSP15 for the cluster representatives produced from the NSP15-ZINC000104379474 complex second replicate.*

***Supplementary Table S2** shows the different components of MM-GBSA for the* NSP15-ZINC000104379474 complex second replicate.

SYSTEM	ΔVDWAALS	ΔEEL	ΔEGB	ΔESURF	ΔGGAS	ΔGSOLV	ΔTOTAL	-TΔS	ΔG BINDING
**NSP15-ZINC000104379474 COMPLEX**	-32.94 ± 3.69	-204.27 ± 19.12	215.65 ± 14.01	-4.61 ± 0.29	-237.21 ± 18.04	211.04 ± 13.94	-26.17 ± 6.89	17.92 ± 3.14	-8.25 ± 7.57

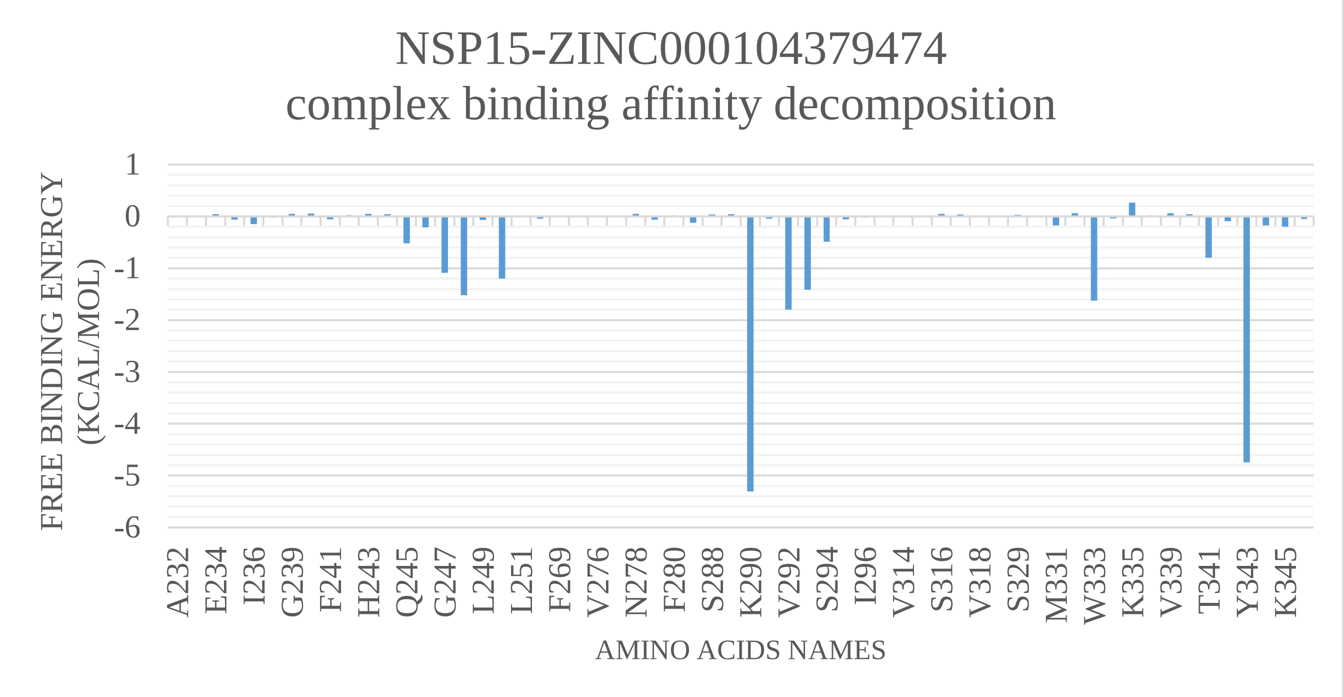

Supplementary Figure S4 *shows the binding free energy decomposition for the NSP15-ZINC000104379474 complex second replicate. Eight amino acids show a contribution of less than -1 Kcal/Mol. G247 (-1.08 Kcal/Mol), G248 (-1.51 Kcal/Mol), H250 (-1.19 Kcal/Mol), K290 (-5.3 Kcal/Mol), V292 (-1.8 Kcal/Mol), C293 (-1.41 Kcal/Mol), W333 (-1.62 Kcal/Mol), and Y343 (-4.74 Kcal/Mol) are the eight amino acids and their contribution.*
